# The dual role of tetraspanin CD63 in HIV-1 replication

**DOI:** 10.1186/1743-422X-11-23

**Published:** 2014-02-08

**Authors:** Guangyu Li, Mark A Endsley, Anoma Somasunderam, Sonia L Gbota, Maryann I Mbaka, James L Murray, Monique R Ferguson

**Affiliations:** 1 Department of Internal Medicine, Division of Infectious Diseases, School of Medicine, University of Texas Medical Branch, Galveston, Texas 77555-0435, USA; 2 GeneTAG Technology, Inc, 3155 Northwoods Place, Norcross, GA 30071, USA

**Keywords:** HIV-1 replication cycle, Tetraspanin CD63, CD4^+^ T cells, Macrophages

## Abstract

**Background:**

Previously, we showed that the tetraspanin membrane protein CD63 mediates both early and post-integration stages of the HIV-1 replication cycle. The temporal roles of CD63 were discerned using monoclonal antibodies and small interfering RNAs (siRNAs) to block CD63 function, and determining which of the sequential steps in HIV-1 replication were disrupted. Inhibition was shown to occur during early infection, suggestive of involvement in virus entry or reverse transcription. In addition, we have shown that treatment with CD63 siRNA post-infection, significantly inhibited virus production in supernatant, suggesting an important role for CD63 in macrophages during HIV-1 replication events occurring after proviral integration, and possibly during egress.

**Results:**

In this study we used CD63 siRNA to investigate the infectivity of pseudotyped viruses (carrying an NL4-3 Env-negative luciferase backbone) in primary human macrophages. We demonstrated that lab adapted R5- and R5X4-tropic HIV-1 strains are significantly inhibited by CD63 silencing. However, the infectivity of MLV or VSV-pseudotyped strains, which enter though receptor-mediated endocytosis, is unaffected by silencing CD63. These results indicate that CD63 may support Env-mediated entry or fusion events facilitated though CD4 and CCR5. Also, antibody and siRNA-based CD63 inhibition studies indicate a potential role for CD63 following proviral integration. Further, we show that CD63 expression is key for efficient replication in primary CD4^+^ T cells, complementing our prior studies with primary human macrophages and immortalized cell lines.

**Conclusions:**

Collectively, these findings indicate that CD63 may support Env-mediated fusion as well as a late (post-integration) step in the HIV-1 replication cycle.

## Background

Macrophages (MØ) and CD4^+^ T lymphocytes are target reservoirs for HIV-1 infection and replication, and play critical roles in multiple aspects of viral pathogenesis. HIV-1 relies on numerous host determinants for successful completion of each of the replicative steps of the viral life cycle from entry to egress [1-11].

CD63 is a type II cellular membrane protein belonging to the tetraspanin superfamily [12,13]. CD63 associates with tetraspanin-enriched microdomains (TEMs), which have been shown to act as gateways for HIV-1 budding at the plasma membrane [14]. Numerous studies have shown that CD63 readily incorporates into HIV-1 virions [15-17] and partially colocalizes with HIV-1 Env and Gag proteins in HIV-1 producing cells [16,18-21]. Colocalization and virion incorporation data indirectly suggest a positive role for CD63 in the HIV-1 replication cycle, although this has not always been borne out experimentally, and conflicting results have been observed. For example, a recent study showed that CD63 suppresses trafficking of the chemokine receptor CXCR4 to the cell surface, which affects HIV-1 entry into T lymphocytes [22-24]. However, we have demonstrated that anti-CD63 monoclonal antibody or siRNA treatment does not affect the expression of the other major coreceptor CCR5, or the primary receptor CD4 [25-27]. Another recent paper showed CD63 is not required for HIV-1 infection of human MØ [28], in contrast with our prior studies.

Our laboratory and others have demonstrated that CD63 is important for HIV-1 replication in primary MØ and CD4^+^ cell lines [25-27,29]. Our earlier studies showed that anti-CD63 monoclonal antibody treatment 30 min prior to and during infection markedly reduced HIV-1 replication in MØ [25]. Inhibition was shown to occur during early infection, suggestive of involvement in virus entry or reverse transcription. Subsequently, we confirmed the requirement of CD63 in HIV-1 replication in primary human MØ and an immortalized CD4^+^ cell line following CD63 down regulation by siRNA [26,27], and presently show its requirement by HIV-1 in primary human CD4^+^ T lymphocytes. Here, we show that CD63 expression supports Env-mediated fusion during MØ transduction with pseudotyped viruses. Down modulating CD63 expression in latently infected U1 cells blocked viral egress, but not intracellular p24 production, indicating CD63 also regulates a late replication step(s). Implications of our findings with regard to mechanisms of HIV-1 replication are discussed below.

## Results and discussion

### CD63 facilitates virus Env-mediated entry in human MØ

**Figure 1:**
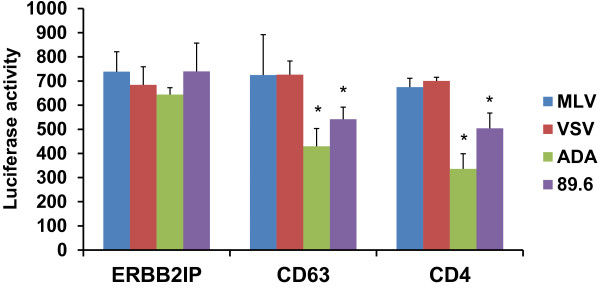
**CD63 facilitates HIV-1 env-mediated entry in human MØs.** Human monocyte derived MØ (5 × 10^5^ cells/well) were transfected with 50 nM siRNAs (CD63, CD4, and ERBB2IP). Forty-eight hours post-transfection, cells were transduced with reporter viruses pseudotyped with various viral Env proteins (m.o.i. = 0.02) that contained the HIV-1 NL4-3 envelope-negative luciferase backbone. Cell lysates were collected on day 3 post-transduction, and luciferase activities were analyzed to monitor pseudovirus integration. MLV and VSV were used as controls for the entry step because they enter the cells via receptor-mediated endocytosis. In contrast, pseudoyped viruses coated with R5 (ADA) and R5/X4 dual-tropic (89.6) HIV-1 Env proteins gain entrance into MØs by sequential Env interactions with CD4 and CCR5, resulting in viral-cell membrane fusion. All experiments were performed in quadruplicate. *P < 0.05, compared with ERBB2IP group.

We studied the effect of silencing CD63 expression on the transduction of various pseudotyped viruses carrying an NL4-3 envelope-negative luciferase backbone. Primary human MØ were transfected with siRNAs (CD63, CD4, or ERBB2IP). The efficiency of CD63-specific siRNA down regulation has been shown in our previous studies [26,27]. Forty-eight hours post-transfection, cells were transduced with various Env-specific reporter pseudoviruses (MLV, VSV, HIV-1_ADA_, or HIV-1_89.6_) for single-cycle infection. Luciferase activities were analyzed 3 days post-infection to monitor pseudovirus integration. MLV and VSV (carrying the same NL4-3 envelope-negative luciferase backbone) were used as controls because they enter the cell via endocytosis, independently of CD4 and chemokine receptor usage. ERBB2IP siRNA was used as a cellular target negative control, as our previous studies showed that silencing ERBB2IP does not inhibit HIV-1 [30,31]. Figure 1 shows that when viral entry is mediated though CD4 and coreceptor CCR5 using R5 (HIV-1_ADA_) or dual-tropic (HIV-1_89.6_) pseudotyped envelopes, infectivity is significantly reduced in CD63-silenced MØ; consistent with the CD4 siRNA control. However, when viral entry is redirected though the endocytic pathway by pseudotyping virions with MLV or VSV, infectivity is not affected in CD63- or CD4-silenced MØ.

### CD63 mRNA down regulation by siRNA in human peripheral blood lymphocytes (PBLs)

Like human MØ, CD4^+^ T lymphocytes are also target reservoirs for HIV-1 infection and replication. In our previous studies we showed that CD63 silencing affects HIV-1 production in monocyte-derived MØ and a CD4^+^ cell line [25-27], and we now extend our investigations to study the role of CD63 in primary human peripheral blood lymphocytes (PBLs).

**Figure 2:**
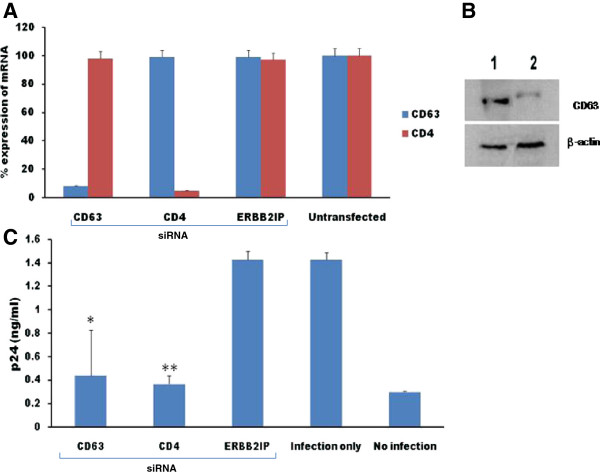
**Silencing CD63 expression in human PBLs by RNA interference and its effect on HIV-1 replication. (A)** PBLs (1 × 10^5^  cells/well) were plated in triplicate in 24-well plates and transfected with 50 nM siRNAs (CD63, CD4, and ERBB2IP). Controls included untreated cells. Total mRNA was isolated from each well using the Qiagen RNeasy kit 48 h after transfection. Quantitative RT-PCR was used to determine relative CD63 expression levels, normalizing to GAPDH expression as an internal control. CD63 expression in non-transfected cells was set to 100%. **(B)** Western blot analysis was performed using cell lysates obtained from PBLs 48 h after transfection. The blot was probed with anti- human CD63 antibody, or an anti-β-actin antibody as an internal control for cellular protein expression. The upper panels of lanes 1 and 2 show the respective expression of CD63 in untreated cells and in CD63 siRNA transfectants. **(C)** PBLs (1 × 10^5^ cells/well) were plated in triplicate in 24-well plates and transfected with 50 nM siRNAs (CD63, CD4, and ERBB2IP). Forty-eight hours post-transfection, cells were infected with HIV-1_89.6_ (m.o.i. = 0.02). Supernatants obtained from PBLs were harvested for p24 detection on day 7 post-infection using p24 Capture ELISA kit (ImmunoDiagnostics, Woburn, MA). *P < 0.05, **P < 0.01, compared with ERBB2IP control group, respectively.

Efficient silencing of CD63 mRNA expression in PBLs with siRNA was confirmed by quantitative reverse transcriptase PCR (qRT-PCR). CD63 mRNA expression was reduced by >92% following CD63 siRNA transfections of PBLs (Figure 2A). CD63 mRNA was not significantly reduced by control siRNA transfections (CD4, ERBB2IP) indicating specificity for CD63 siRNA down regulation in PBLs. Western blot analysis revealed that CD63 protein expression (53 kDa) was significantly reduced in CD63-siRNA transfectants compared to untransfected cells (Figure 2B). Reducing CD63 expression in PBLs did not affect viability, as assayed by coupling GAPDH to 3-phosphoglyceric phosphokinase and measuring ATP production (data not shown).

### CD63 down regulation affects HIV-1 production in human PBLs

We sought to elucidate the effect of CD63 down regulation on viral production in PBLs. PBLs were transfected with CD63 siRNA or control siRNAs (ERBB2IP or CD4) for 48 h, followed by infection with HIV-1_89.6_. HIV-1 production was assessed in culture supernatants by p24 ELISA at 7 days post-infection. As shown in Figure 2C, virus production in PBLs was significantly reduced following CD63 or CD4 silencing compared to ERBB2IP siRNA transfected cells (P < 0.05). Thus, our data strongly suggest that CD63 expression supports HIV-1 replication in PBLs.

### CD63 down regulation also affects later events of the HIV-1 replication cycle

**Figure 3:**
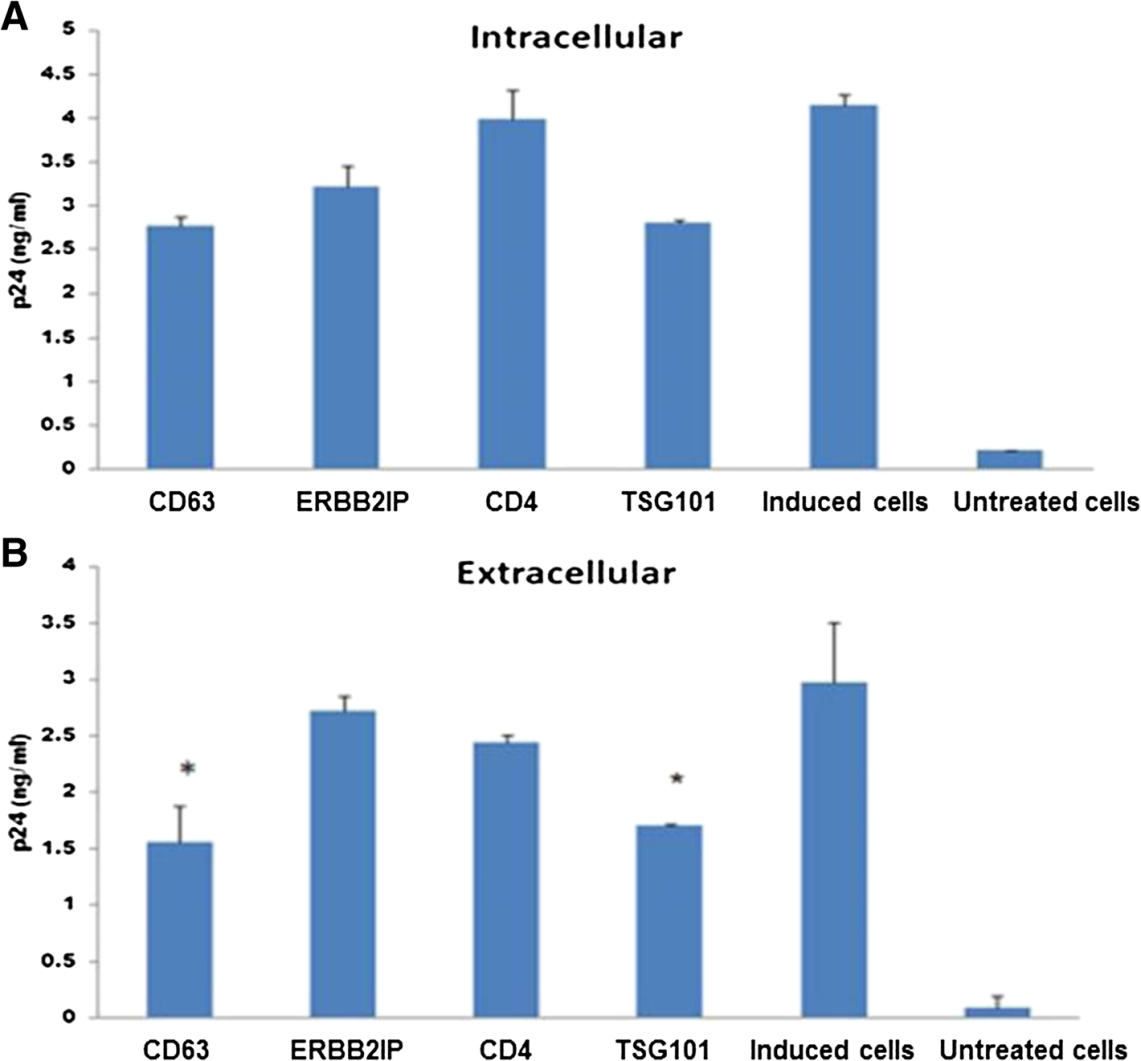
**CD63 down regulation affects later events of the HIV-1 replication cycle.** U1/HIV-1 cells (5 × 10^5^ cells/well) were transfected with CD63-, ERBB2IP-, CD4-, or TSG101-specific siRNA. Two days post-transfection, cells were induced with 3 phorbol 12-myristate 13-acetate (PMA, 5 × 10^-6^ M). Three days post-induction, HIV-1 p24 protein in cell lysates **(A)** and culture supernatants **(B)** were detected using a p24 Capture ELISA kit (ImmunoDiagnostics, Woburn, MA). TSG101-specific siRNA was used as a control because it is involved in post-integration trafficking, assembly, and release of HIV-1 Gag proteins [34]. Experiments were performed in triplicate. *P < 0.05, compared to ERBB2IP group.

To further assess the role of CD63 in late stage HIV-1 replication, CD63 siRNA was transfected into U1/HIV-1 cells, which are chronically infected monocytoid cells harboring 2 integrated copies of provirus per cell [32,33]. Early steps of HIV-1 replication (such as entry and reverse transcription) are not required for replication in these cells, and virus production can be induced with 3 phorbol 12-myristate 13-acetate (PMA). Silencing CD63 did not significantly inhibit intracellular p24 production (Figure 3A), suggesting that CD63 does not regulate viral p24 protein production. However, extracellular HIV-1 p24 obtained from supernatants was significantly reduced in CD63- and TSG101-depleted U1 cells (p < 0.05) compared to the ERBB2IP siRNA control (Figure 3B). TSG101 is involved in post-integration trafficking and release of HIV-1 Gag proteins [34]. Similar to TSG101, these findings indicate that CD63 may also serve a role in the release of HIV-1 from the cell, which may relate to why HIV-1 readily incorporates CD63 into nascent virions. These outcomes are also consistent with our previous studies performed in primary MØ. Treatment with CD63-specific siRNA 3 days post-infection, significantly inhibited virus production in culture supernatants [26], suggesting an important role for CD63 in MØ during HIV-1 replication events occurring after proviral integration, and possibly during egress.

Previously, we have shown that CD63 silencing significantly inhibits HIV-1 replication in MØ [25,27], U1/HIV-1 producing cells [26], U373 CD4^+^ cell line [26], and presently in T lymphocytes (Figure 2C), revealing that CD63 expression is broadly required for replication in relevant HIV-1 target cells.

We further demonstrate dual roles for CD63, showing that it contributes to both early and late events of the HIV-1 replication cycle. CD63 mediated efficient entry of HIV-1 Env pseudotyped viruses into CCR5^+^ MØ (Figure 1). When viral entry was redirected though the endocytic pathway by pseudotyping virions with MLV or VSV, infectivity in MØ was not affected by CD63 silencing. However, CD63 silencing did reduce infectivity with R5- and R5X4-tropic viral pseudotypes that depend on CD4 and CCR5 for entry. This indicates (i) that CD63 supports Env-mediated entry or fusion events facilitated though CD4 and CCR5, and (ii) that the entry events are specific for the HIV-1 Env protein. In support, other investigators have demonstrated that the extracellular domain 2 of CD63 partially colocalizes with CD4 at the cell surface of macrophages [35].

CD63 also exerts a role in later stages of the HIV-1 replication cycle. As CD63 is a major component of late endosomes, it is possible that CD63 is also involved in HIV-1 maturation and budding in MØ. In this study, we show that silencing CD63 expression decreases extracellular, but not intracellular, HIV-1 p24 production (Figure 3A, B), indicating that CD63 may influence viral egress. In MØ, nascent HIV-1 virions accumulate and assemble in multi-vesicular bodies (MVBs) or endosomes containing tetraspanins such as CD63 and CD81, and may be released subsequently as exosomes. However, in T-cells HIV-1 Gag assembly is localized to the plasma membrane [14] where CD63 is also found. As CD63 is located in endosomes and the plasma membrane where HIV assembly takes place, it is possibly involved in virus assembly at these sites. This complex localization pattern of CD63 suggests that its intracellular trafficking and distribution must be tightly regulated.

Several studies have shown that CD63 supports HIV-1 replication [25-27], is located at assembly initiation sites [36-38], and is incorporated into nascent virions [15,16], yet viral binding partners with CD63 have remained elusive. Exactly how CD63 is targeted to viral assembly sites and incorporated into budding HIV-1 particles also remains unclear. A plausible explanation is that virus budding may occur in TEMs and that CD63 must be present for the completion of the recycling loop with return of both CD63 and viral proteins to a common site for virus release. CD63-containing TEMs may be required for budding of virus from these cells.

## Conclusions

In conclusion, we have shown that CD63 plays a role both in the early entry stages of the HIV-1 replication cycle in concert with CD4 and/or CCR5, and in a late replicative step, possibly at the level of egress. Further research is needed to gain insight into the molecular mechanisms of CD63 that facilitate early and late events in the HIV-1 replication cycle.

## Methods

### Cells and culture conditions

Primary monocyte-derived macrophages (MØ) and peripheral blood lymphocytes (PBLs) were isolated and purified from buffy coats of healthy HIV-negative blood donors prepared by the UTMB Blood Bank (Galveston, TX). Monocyte-derived MØ were prepared by Ficoll-Hypaque gradient centrifugation followed by adherence for 7 days to plastic petri dishes. During differentiation, MØ were cultured in Iscove’s modified Dulbecco’s medium (IMDM) [39] supplemented with 20% FCS; 1% L-glutamine and 1% Penicillin/Streptomycin as described in [39]. Nonadherent cells (PBLs) were treated with phytohemagglutinin (PHA) for 72 h 37°C with 5% CO_2_ prior to propagation in RPMI 1640 supplemented with 20% fetal calf serum (FCS), 20 units/ml IL-2, 1% Penicillin/Streptomycin.

Our experimental research has been approved by the Institutional Biosafety Committee (IBC) at the University of Texas Medical Branch, Galveston, Texas. The IBC reference numbers are 2013040 (for use of HIV) and 2012033 (for use of primary cells and cell lines). There is no use of animals or humans in our studies.

U1/HIV-1 cells [34] (1 × 10^6^) obtained from the NIH AIDS Research and Reference Reagent Program were plated in 24-well microtiter plates on day 1. Complete cell media (RPMI 1640 containing 2.0 mM L-glutamine; 10% heat-inactivated fetal bovine serum) was changed after 24 h. Forty-eight hours post-transfection (day 3), complete RPMI media plus phorbol 12-myristate 13-acetate (PMA) was added to cells. Intracellular and extracellular virus was measured in the cells lysates and culture supernatant on day 5 by p24 ELISA.

### Preparation of HIV-1_89.6_

Dual-tropic (R5/X4) HIV-1_89.6_ is an HIV-1 laboratory adapted strain originally isolated from an infected individual. The original preparation was prepared from molecularly cloned virus, and grown in CEMx174 cells [40] and donated to the NIH AIDS Research and Reference Reagent Program. We purchased HIV-1_89.6_ from the Virology Core Facility, Center for AIDS Research at Baylor College of Medicine, Houston, TX, which was prepared and propagated in human PBMCs. HIV-1_89.6_ stock containing 49.977 ng/ml of HIV p24 with 261,300 TCID50/ml was used to infect PBLs at an m.o.i of 0.02.

### siRNA transfections and infectivity assay

siRNAs targeting CD63, CD4, ERBB2IP, and TSG101-specific were designed and synthesized by Dharmacon (Lafayette, CO). The target sequences for siGENOME SMARTpool siRNA of CD63 includes D-017256 (01-GAGAUAAGGUGAUGUCAGA; 02-AAGGAGAACUAUUGUCUUA; 03-GGAUUAAUUUCAACGAGAA; 04-GAUGGAGAAUUACCCGAAA). The target sequences for siGENOME SMARTpool siRNA of CD4 includes D-005234 (01-GAACUGACCUGUACAGCUU; 02-AAUCAGGGCUCCUUCUUAA; 03-GAAGAAGAGCAUACAAUUC; 18-AUUACCAAGUGCCGGACUA); The target sequences for siGENOME SMARTpool siRNA of TSG101 includes M-003549 (01-AAACUGAGAUGGCGGAUGA; 02-GAACCUCACUGGAACAAUC; 04-CCGUUUAGAUCAAGAAGUA; 05-UCCCACAGCUCCCUUAUAC). The target sequences for siGENOME SMARTpool siRNA of ERBB2IP includes D-031861 (01-UGAAACAGCUCACAUAUUU; 02-UGUGAAAUCUCAUAGCAUA; 03-CGAAGAGCCAAAUAUAAUA; 04-CCAAACGACCGACUUAUUC). siRNAs transfections were performed using oligofectamine (Invitrogen, CA) or the Nucleofector™ Technology by Amaxa (Lonza, Switzerland) according to the manufacturers’ instructions. In brief, MØ adherent for 5 days were plated in 24-well plates (5 × 10^5^ cells/well), and transfected with 50 nM siRNAs using oligofectamine in serum-free Opti-MEM I medium (Gibco) 24 h after plating. MØ were maintained in Iscove’s medium and 20% FCS after 4 h to terminate transfection. U1/HIV-1 cells were transfected with siRNA (200 nM final concentration) using Oligofectamine (Invitrogen) following the manufacturer’s instructions.

In order to measure down regulation of target genes in PBLs, cell lysates were prepared with cell lysis buffer (Promega, WI) at 2 days post-transfection, and were collected for Western blot analysis, or RNA was extracted for real time quantitative PCR analysis. PBLs were transfected with 50 nM siRNAs in 6-well plates (5 × 10^5^ cells/well) using Nucleofector^®^, and standard applications of manufacture’s protocol (Amaxa) were followed. For Western blot analysis, anti-human CD63 monoclonal antibody (Santa Cruz) was used.

Forty eight hours post-transfection, PBLs were infected with HIV-1_89.6_ (m.o.i. = 0.02). Culture supernatants were harvested for p24 detection on day 7 post-infection using a p24 Capture ELISA kit (ImmunoDiagnostics, Woburn, MA).

### HIV-1 Env pseudovirus production and titration

Pseudotype viruses containing the luciferase gene provide an efficient way to measure infection after CD4, CCR5, and CD63 down regulation. Stocks of single-round-infection of various viral Env pseudoviruses (MLV, VSV, HIV-1_ADA_, or HIV-1_89.6_) were produced by cotransfecting 293 T cells (1.7 × 10^7^ cells per T75 flask) with 2 μg of an HIV-1 rev/env expression plasmid and 12 μg of an env-deficient HIV-1 backbone plasmid (pNL4.3 ΔEnv) using Lipofectamine transfection reagent (Invitrogen). The NL4-3 proviral clone has a luciferase in place of *nef* (*env*^*-*^*luc*). Pseudovirus-containing supernatant was harvested 24 h following transfection and clarified by centrifugation and 0.45-μm filtration. Single-use aliquots (1.0 ml) were stored at −80°C. The 50% tissue culture infectious dose (TCID_50_) for each pseudovirus preparation was determined by infection of TZM-bl cells. These pseudovirus stocks then were used for transduction of MØs.

### Real time quantitative PCR

Total mRNA was isolated from siRNA-transfected cells and MØ using RNeasy Mini Kits (Qiagen). CD63 specific primers and probe were purchased from Applied Biosytems (Carlsbad, CA). All reactions were performed using Applied Biosystems TaqMan Universal Master Mix and run using an Applied Biosystems 7500 Fast Real Time PCR system and 7500 Fast System Software. Silencing of target genes was determined by normalizing target gene expression to GAPDH expression (n = 3).

### Statistical analysis

The results are expressed as mean ± SD of at least four wells. Two-tailed, paired Student’s *t-*test was used to determine statistical significance. P values of <0.05*, and <0.01** were considered significant.

## Competing interests

The authors declare that they have no competing interests.

## Authors’ contributions

GL and MAE performed CD63 molecular studies for early and late events of the HIV-1 replication cycle and drafted the manuscript. AS, SLG, MIM performed studies to investigate the role of CD63 in HIV-1 infected human peripheral blood lymphocytes. JLM participated with design of the study and contributed to drafting the manuscript. MRF conceived of this study, participated in its design and coordination, and contributed in drafting the manuscript. All authors have read and approved the final manuscript.
